# [^18^F]FDG PET during immunotherapy: mind the gap between evidence, imaging guidelines, and oncology practice

**DOI:** 10.1007/s00259-026-08080-1

**Published:** 2026-07-17

**Authors:** Giulia Santo, Chiara Pozzessere, Olivier Michielin, Giuseppe Lucio Cascini, John O. Prior, Francesco Cicone

**Affiliations:** 1 Nuclear Medicine Unit, Department of Experimental and Clinical Medicine, “Magna Graecia” University of Catanzaro, Campus “Salvatore Venuta”, Viale Europa, Catanzaro, 88100 Italy; 2 Department of Diagnostic and Interventional Radiology, Lausanne University Hospital, Lausanne, Switzerland; 3 Precision Oncology, University Hospital of Geneva (HUG), Geneva, Switzerland; 4 Department of Medicine and Heath Sciences “Vincenzo Tiberio”, University of Molise, Campobasso, Italy; 5 Department of Nuclear Medicine and Molecular Imaging, Lausanne University Hospital, Lausanne, Switzerland

**Keywords:** Melanoma, Non-Small Cell Lung Cancer, Positron Emission Tomography Computed Tomography, Immune Check Point Inhibitors, Treatment monitoring

## Abstract

No specific statements regarding the use of 2-[^18^F]fluoro-2-deoxy-D-glucose positron-emission tomography/computed tomography ([^18^F]FDG PET) in the context of immunotherapy are made in current international oncological guidelines for the management of the two cancer types for which the largest body of evidence has been generated, such as melanoma and non-small cell lung cancer (NSCLC). However, practice guidelines released jointly by leading nuclear medicine societies recommended the use of [^18^F]FDG PET alongside computed tomography (CT) before immunotherapy initiation, for early response assessment, and in patients considered for therapy discontinuation. In the present paper we critically reviewed the available literature on the comparison between [^18^F]FDG PET and CT for response assessment in patients undergoing immunotherapy, with the aim to identify where metabolic imaging holds the most significant promise. In early response assessment, where an accurate discrimination between pseudoprogression and true tumor progression is necessary, [^18^F]FDG PET is of limited value. In contrast, there are at least two clinical scenarios in which [^18^F]FDG PET could outperform CT because of its high negative predictive value, that are the evaluation of response to neoadjuvant immunotherapy or before therapy discontinuation. We emphasized that the generation of robust evidence is necessary before clinical implementation, as it ensures the optimal allocation of resources and better patient care.

## Introduction

Imaging modalities such as computed tomography (CT) and 2-[^18^F]fluoro-2-deoxy-D-glucose positron emission tomography/computed tomography ([^18^F]FDG PET) have strengths and limitations that should be carefully considered depending on the clinical context in which they are used. For most solid tumors, treatment response on CT is assessed using the Response Evaluation Criteria in Solid Tumors (RECIST) criteria, current version RECIST 1.1 [1]. These are size-based criteria with typical limitations in the interpretation of changes in contrast enhancement and in the assessment of tumor necrosis, which may lead to an underestimation of treatment responses in absence of significant tumor shrinkage [2–4]. On the other hand, functional imaging with [^18^F]FDG PET often enables an earlier detection of tumor recurrences or of tumor responses, but its higher sensitivity is generally counterbalanced by high rates of false positive findings. In the last decades, a paradigm shift has been observed from the use of classical cytotoxic therapies towards the introduction of novel anticancer agents targeting specific molecular pathways or blocking cancer-induced immunosuppression. These newer oncological therapies may profoundly affect the interpretation of imaging studies, depending on tumor type and on treatment mechanisms [5, 6]. Among the most relevant innovations, therapy with immune checkpoint inhibitors (ICI) led to substantial improvements of survival rates of patients with various solid malignancies [7–9], at the same time representing a challenge for medical imaging due to the emergence of previously unrecognized response patterns. Among these patterns, the phenomenon of pseudoprogression is particularly relevant, occurring in up to 10% of patients with differences in incidence rates depending on cancer and ICI types [10]. Pseudoprogression was first described by Di Giacomo et al. in a patient with metastatic melanoma treated with anti-CTLA-4 [11] and is characterized by an enlargement of known tumor lesions and/or by the appearance of previously undetectable tumor lesions which subsequently regress or disappear later during immunotherapy [12]. This can be explained by tumor infiltration by immune cells, such as CD4 T cells, CD8 T cells and macrophages [13], as well as by the presence of tumor necrosis, hemorrhage and edema, and represents an initial response to treatment [11, 14]. On imaging, pseudoprogression may be misinterpreted as real tumor growth and incorrectly classified as progressive disease (PD).

To account for pseudoprogression, the modified RECIST for immune-based therapeutics (iRECIST) introduced the concept of unconfirmed progressive disease. Accordingly, lesions appearing early after treatment initiation are evaluated separately from initial target and non-target lesions, and the definition of PD requires additional imaging following at least 4 weeks to confirm a further increase in the size or in the number of suspect lesions [15]. The use of metabolic imaging with [^18^F]FDG PET has also been proposed in the workflow of patients treated with immunotherapy, particularly for treatment response evaluation [16, 17].

In 2022, a joint document authored by the leading nuclear medicine societies of Europe, USA and Australia —titled “Joint EANM/SNMMI/ANZSNM Practice Guidelines/Procedure Standards on the Recommended Use of [^18^F]FDG PET/CT Imaging During Immunomodulatory Treatments in Patients with Solid Tumors, Version 1.0”—was released [18]. In this document it is defined as essential to perform a baseline [^18^F]FDG PET scan before initiating immunotherapy to ensure accurate tumor assessment and to provide a basis for tumor monitoring. Additionally, the authors recommended that a follow-up [^18^F]FDG PET is conducted 8–12 weeks after treatment initiation (i.e., following 3–4 cycles of therapy) for early response assessment, as well as to complement the information obtained from morphological imaging with CT for a correct classification of inconclusive findings. The use of [^18^F]FDG PET scan was also suggested later during the treatment course to confirm the absence of residual metabolically active disease in patients requiring therapy discontinuation, particularly those showing long-term partial response or stable disease on CT. Lastly, in patients who temporarily withdrawn from treatment, [^18^F]FDG PET was recommended before restarting therapy to establish a new baseline for subsequent response assessment.

The present work had two major aims. Firstly, by analyzing the recommendations of major oncological guidelines, we sought to determine what the state of the art is regarding the implementation of [^18^F]FDG PET during immunomodulatory treatments in the two cancer types for which the largest body of evidence has been generated [7–9, 19, 20], such as melanoma and non-small cell lung cancer (NSCLC). In second instance, we systematically reviewed the available evidence on the comparison between [^18^F]FDG PET and CT for the evaluation of response to immunotherapy in patients with these tumors. The implications of these analyses are discussed, key priorities for future research are identified, and a methodology for the design of clinical trials evaluating the role of imaging during immunotherapy is presented.

### The role of [^18^F]FDG PET for treatment monitoring and response evaluation: analysis of oncology practice guidelines for melanoma and NSCLC

We searched the most authoritative international oncological guidelines, including those of the European Society for Medical Oncology (ESMO), the National Comprehensive Cancer Network (NCCN), and the Cancer Council of Australia (CCA), to look for specific recommendations on the use of [^18^F]FDG PET for treatment monitoring and response evaluation in cancers commonly treated with immunotherapy, such as melanoma [21–23] and NSCLC [24–28].

Of note, from these documents, it is not generally possible to derive recommendations for the imaging monitoring of different types of anticancer treatments, such as chemo/radiotherapy, targeted therapies or immunotherapies, as all treatments are usually lumped together and/or monitored in the same way.

As summarized in Table 1, none of these major guidelines, including the most recent versions available at the time of writing, explicitly endorse [^18^F]FDG PET as the imaging modality of choice during active anticancer treatment. For the imaging assessment of all systemic anticancer treatments in metastatic NSCLC, CT remains the reference standard in all international guidelines [25–27].

**Table 1:** Indications for the appropriate use of imaging during treatment according to main oncological guidelines for melanoma and NSCLC. Comparison is provided with the recommendations on the use of [^18^F]FDG PET/CT imaging during immunomodulatory treatments in patients with solid tumors released jointly by major nuclear medicine societies

Treatment evaluation:	EANM/SNMMI/ ANZSNM	ESMO		NCCN		CCA	
	All solid tumors under immunotherapy (2022) [18]	Melanoma (2025) [21]	NSCLC (2023) [24–26]	Melanoma (2025) [22]	NSCLC (2026) [27]	Melanoma (2021) [23]	NSCLC (2022) [28]
After neoadjuvant therapy	N/D	N/D	N/D	Cross-sectional imaging* is recommended after 6 to 12 weeks to exclude residual or metastatic disease and assess for surgical planning	Chest CT with or without contrast ± [^18^F]FDG PET should be performed in possibly resectable NSCLC to exclude disease progression or interval development of metastatic disease after induction therapy	N/D	N/D
During Active or Maintenance systemic therapy	[^18^F]FDG PET is recommended at interim, commonly 8–12 weeks (i.e., 3–4 cycles) after treatment start, to complement information obtained from morphological imaging with CT and to resolve discordant findings [^18^F]FDG PET scan can be also performed earlier or later during the course of treatment in case of clinical deterioration and/or suspicious progression on contrast-enhanced CT In patients receiving maintenance therapy or undergoing long-term treatment with ICI, [^18^F]FDG PET may be obtained to assess metabolic response, particularly in partial responders or stable disease on CT	Stage IIB – IV NED: CT (or [^18^F]FDG PET) of neck, thorax, abdomen, pelvis and brain MRI every 3–6 months Stage IV (distant metastases): CT (or [^18^F]FDG PET) monitoring individualized to each patient, depending on therapy and symptoms For patients achieving CR, CT (or [^18^F]FDG PET) staging every 12 weeks after ICI discontinuation	Response evaluation after two to three cycles (or 8–12 weeks) of systemic therapy is recommended using the same initial radiographic investigation that demonstrated tumor lesions Measurements and response assessment should follow Response Evaluation Criteria in Solid Tumours (RECIST) v1.1 Follow-up with [^18^F]FDG PET is not routinely recommended, due to its high sensitivity and relatively low specificity	Monitoring is appropriate and should include clinical examination and imaging (Cross-sectional imaging*±brain MRI) at clinically appropriate intervals (e.g., every 2–6 months) in: (i) stage III local satellite/in-transit disease; (ii) nodal disease in previously dissected nodal bed that is unresectable or incompletely resected; (iii) limited (resectable) distant metastatic disease; (iv) disseminated (unresectable) distant metastatic disease	Monitoring is recommended during initial therapy with CT with or without contrast, of known or high-risk sites of disease after 2 cycles and then every 2 to 4 cycles** During maintenance or subsequent therapy, monitoring is also recommended with CT, with or without contrast every 6 to 12 weeks**	N/D	N/D

*Cross sectional imaging studied include chest/abdomen/pelvis (and neck if clinically indicated CT with intravenous contrast and/or whole-body [^18^F]FDG PET/CT. Choice of modality depends on clinical circumstances. PET may be more sensitive than CT for the detection of distant metastases, particularly in the extremities. **FDG PET not mentioned. Note: CR = complete response; NED = non evident disease; NSCLC = non-small cell lung cancer; N/D = not defined, refers to the use of [^18^F]FDG PET/CT

In non metastastic patients with NSCLC potentially eligible for surgical resection, the NCCN guidelines consider the use of [^18^F]FDG PET for the evaluation of response to neoadjuvant therapy in addition to chest CT [27].

As regards patients with melanoma, the use of [^18^F]FDG PET for monitoring anticancer therapies including immunotherapy is considered together with CT in all guidelines, though it is left at the discretion of individual clinicians without a formal consensus on the timing and the clinical situation in which it would be most useful [21, 22]. Similarly, as for NSCLC, [^18^F]FDG PET is considered by NCCN guidelines as additional or alternative modality to CT in the neoadjuvant setting in patients with melanoma eligible for surgical resection [22]. The CCA guidelines do not specifically refer to imaging monitoring during active treatment for either melanoma or NSCLC [23, 28].

### Review of the literature data on the comparison between [^**18**^F]FDG PET and CT during immunotherapy in melanoma and NSCLC

A systematic analysis of the current literature was performed to compare [^18^F]FDG PET with CT for the evaluation of response to immunotherapy in patients with melanoma and NSCLC, the two cancer types with the largest body of evidence on this topic. A systematic literature search was conducted in PubMed until March 2026. The following search string was used: (“Melanoma“[Mesh] OR melanoma[tiab] OR “Carcinoma, Non-Small-Cell Lung“[Mesh] OR NSCLC[tiab] OR “non small cell lung cancer“[tiab]) AND (“Immune Checkpoint Inhibitors“[Mesh] OR immunotherapy[tiab] OR “immune checkpoint inhibitor*“[tiab] OR ICIs[tiab] OR nivolumab[tiab] OR pembrolizumab[tiab] OR atezolizumab[tiab] OR durvalumab[tiab] OR ipilimumab[tiab]) AND (“Fluorodeoxyglucose F18“[Mesh] OR FDG[tiab] OR “[18F]FDG“[tiab] OR “Positron-Emission Tomography“[Mesh] OR PET[tiab] OR “PET/CT“[tiab]) AND (“Tomography, X-Ray Computed“[Mesh] OR CT[tiab] OR “computed tomography“[tiab]) AND (“Treatment Outcome“[Mesh] OR “therapy response“[tiab] OR “response evaluation“[tiab] OR “response assessment“[tiab] OR “treatment response“[tiab]) AND (compar*[tiab] OR “head-to-head“[tiab] OR versus[tiab] OR vs[tiab] OR “diagnostic performance“[tiab] OR accuracy[tiab]). The literature search retrieved 101 articles. Additional articles were identified through manual screening of the reference lists. Reviews, case reports, editorials, abstracts as well as duplicate studies were excluded. Study selection was performed in accordance with the Preferred Reporting Items for Systematic Reviews and Meta-Analyses (PRISMA) guidelines. Eighteen articles were ultimately selected for review. The clear specification of the use of total-body (i.e., including chest, abdomen and pelvis) contrast-enhanced CT for response evaluation was not a requirement to be included in the analysis.

## Results

**Fig. 1:**
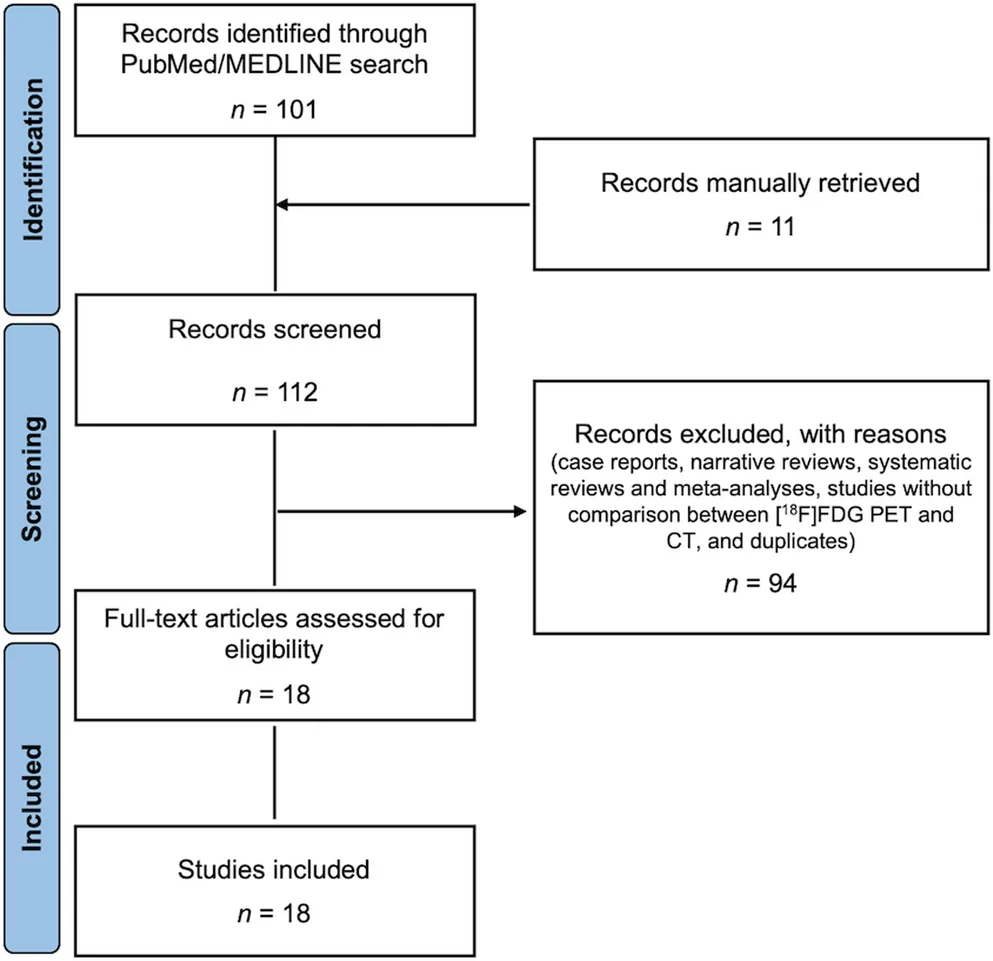
PRISMA flow diagram of the study selection process. A total of 112 records were identified through database searching (PubMed/MEDLINE) and manual searching. After screening, 94 records were excluded for predefined reasons (including case reports, narrative reviews, systematic reviews and meta-analyses, lack of comparison between [^18^F]FDG PET and CT, and duplicates). Eighteen full-text articles were assessed for eligibility, and all were included in the final analysis

The PRISMA flow diagram of the study selection process is shown in Fig. 1. A summary of the major findings of the selected studies is shown in Table 2, together with a grading of the quality of published data according to the Oxford levels of evidence [29]. From the literature search, 18 studies were selected, the majority of which were retrospective (*n* = 13). No phase III trials directly comparing [^18^F]FDG PET and CT imaging in patients receiving immunotherapy were found. In the sections below, dedicated to melanoma and NSCLC, respectively, we provided a more detailed summary of the studies comparing [^18^F]FDG PET and CT for response assessment during immunotherapy included in the analysis. Each section is structured in the same way, i.e. describing first the studies on early response assessment (within the first 12 weeks after treatment initiation), then the studies on delayed response assessment (after 12 months) and, lastly, the studies on response assessment at the end of neoadjuvant immunotherapy.

**Table 2:** Characteristics of the studies comparing [^18^F]FDG PET and CT in the evaluation of response to immunotherapy

	Setting	First author, year, [ref]	Study design	No pts	Study Outcome	Best outcome predictor	Level of evidence*
Melanoma	Neoadjuvant	Zhou et al.2025 [39] †	R	115	Pathological response, RFS	PET	3
	Early response evaluation	Cho et al. 2017 [30] ‡	P	20	Clinical benefit	CT	2
		Amrane et al. 2019 [31]	R	37	PFS, OS	EQUAL	3
		Annovazzi et al. 2020 [50] †	R	57	Clinical benefit, PFS	EQUAL	3
	Late response assessment	Kong et al. 2016 [36]	R	27	BOR	CT	3
		Tan et al. 2018 [32] Dimitriou et al. 2022 [33]	R	104	PFS	PET	3
					OS	EQUAL	
		Mesnard et al. 2020 [35] †	R	26	PFS	PET	3
		Ferdinandus et al. 2022 [34]	R	38	TTP	PET	3
**NSCLC**	Neoadjuvant	Cheng et al. 2025 [51] †	R	131	DFS	PET	3
	Early response evaluation	Beer et al. 2019 [41]	P	42	PFS, OS	EQUAL	2
		Castello et al. 2020 [42]	P	35	PFS	EQUAL	2
					OS	PET	
		Rossi et al. 2020 [43]	P	48	OS	CT	2
		Park et al. 2020 [47] †	R	24	BOR	EQUAL	3
		Kitajima et al. 2021 [49]	R	40	PFS, OS	EQUAL	3
		Ayati et al. 2021 [48] ‡	R	72	PFS, OS	EQUAL	3
		Kaira et al. 2023 [44] †	P	54	PFS, OS	PET	2
		Kitajima et al. 2025 [46]	R	33	PFS, OS	PET	3

In the studies marked with † the use of total-body contrast-enhanced CT for response evaluation was not clearly specified. The two studies marked with ‡ used both contrast-enhanced and non-contrast enhanced CT for response evaluation. In all other studies the use of total-body contrast-enhanced CT for response evaluation was clearly specified. Notes: BOR = Best overall response rate; Clinical benefit = defined as complete or partial response at 4-month or stable disease lasting at least 6 months; EQUAL = no clear advantage of one imaging modality over the other; P = prospective; R = retrospective; OS = overall survival; PFS = progression-free survival; RFS = recurrence free survival; DFS = disease-free survival; TTP = time to progression

*Oxford evidence level 1: randomized controlled trial

Oxford evidence level 2: prospective cohort study or ecological study/outcomes research

Oxford evidence level 3: retrospective cohort study or individual case-control study

### Melanoma

The first prospective comparative imaging study on early response assessment to ICI was conducted by Cho et al. in 2017 on 20 patients with metastatic melanoma [30]. Tumor responses were analyzed using [^18^F]FDG PET and CT after 3–4 weeks and after 4 months of treatment. At the earliest time point, the agreement in tumor response assessment between the two imaging modalities was relatively low because of a higher number of incorrect [^18^F]FDG PET interpretations compared to CT, particularly in evaluating stable disease. Most patients with stable disease at CT and moderately to markedly increased [^18^F]FDG uptake ultimately showed good clinical response rates including complete/partial response or durable stable disease. At a later time point, the agreement between [^18^F]FDG PET and CT interpretation improved significantly, though RECIST 1.1 criteria still confirmed better prediction of clinical response than [^18^F]FDG PET. However, in another study including retrospectively 37 patients with metastatic disease, [^18^F]FDG PET performed 8–12 weeks following treatment initiation proved equal to CT for prediction of progression-free survival (PFS) and overall survival (OS) [31].

Since immunotherapy induces immune cell infiltration and tumor inflammation mainly in the early stages of treatment, a delayed [^18^F]FDG PET scan, performed around one year after treatment initiation, was adopted by Tan and colleagues in 104 patients during active treatment or in patients who had discontinued immunotherapy. In this study, a delayed [^18^F]FDG PET offered more precise prognostic insights compared to CT, largely due to the high negative predictive value of PET, which enabled to correctly classify as complete metabolic responders 49/104 patients who showed only partial response or stable disease at CT [32]. In the long-term analysis of the study, [^18^F]FDG PET confirmed its superior ability to stratify PFS compared to CT-based assessment [33]. Similar results were shown by subsequent smaller retrospective studies including metastatic patients who had discontinued immunotherapy [34, 35]. Yet metabolically active isolated lesions may occasionally become evident in patients with prolonged (i.e., > 12 months) responses to anti-PD-1 therapy. These findings most likely correspond to immune cell infiltrates rather than to residual tumor [36].

More recently, the role of [^18^F]FDG PET in assessing response to neoadjuvant immunotherapy has gained increasing attention. The NeoPele [37] and NeoTrio [38] trials are investigating, as secondary study endpoint, the prognostic value of [^18^F]FDG PET/CT in patients with resectable stage III melanoma undergoing neoadjuvant immunotherapy prior to surgery. A recent post hoc analysis involved 115 stage III melanoma patients who underwent baseline and preoperative [^18^F]FDG PET and CT imaging, followed by surgical resection [39]. The authors found that RECIST 1.1 criteria underestimated pathological response, as patients with stable disease on CT still had a 50% likelihood of achieving a major pathological response (MPR) defined according to the International Neoadjuvant Melanoma Consortium criteria [40]. Conversely, the responses observed with [^18^F]FDG PET better correlated with the obtainment of a MPR. In patients showing complete metabolic response, MPR was as high as 96%. Interestingly, [^18^F]FDG PET better stratified PFS compared to both pathology and CT [39].

In summary, the available literature on the comparison between [^18^F]FDG PET and CT for early assessment of treatment response during immunotherapy in metastatic melanoma is limited and characterized by generally low evidence levels. The available data show no clear advantage of [^18^F]FDG PET over CT in this setting. In fact, [^18^F]FDG PET may lead to a higher number of false-positive findings, particularly early after treatment initiation (3–4 weeks). However, after 8–12 weeks, both [^18^F]FDG PET and CT appear to be equally effective for outcome prediction. The indication for which [^18^F]FDG PET appears to be more accurate than CT is the confirmation of response in patients who are eligible either for treatment discontinuation, or for surgery at the end of neoadjuvant immunotherapy. Future randomized trials should be designed to assess whether a treatment de-escalation strategy can be safely proposed to patients obtaining a complete metabolic response on [^18^F]FDG PET after neoadjuvant immunotherapy.

### NSCLC

Mixed results on the role of early response assessment with [^18^F]FDG PET during active immunotherapy treatment were reported in NSCLC [41–46]. In a study including 35 patients with NSCLC assessed after 3–4 cycles of immunotherapy (8–12 weeks), Castello and colleagues demonstrated that only PET-based criteria were predictive of OS, outperforming CT-based assessment [42]. In contrast, Rossi et al., showed that only CT-based assessment correlated with OS in 48 patients with advanced NSCLC assessed after 4 cycles of immunotherapy [43]. Other studies showed no significant differences between conventional and metabolic response assessment methods in the early differentiation between responders and non-responders to ICI after 3–4 cycles of treatment [47–50].

Same as for melanoma, the use of [^18^F]FDG PET in the neoadjuvant setting is gaining momentum. A study enrolling 131 patients with stage II/III NSCLC who underwent 3 cycles of neoadjuvant chemo-immunotherapy before surgical resection was recently published. The authors correlated the results of [^18^F]FDG PET and those of CT obtained after chemo-immunotherapy with long-term post-surgical follow up. Patients who achieved a complete [^18^F]FDG PET metabolic response after chemo-immunotherapy had a significantly longer disease-free survival than those who had residual disease, whereas the obtainment of a response on CT, either complete or partial, did not significantly stratify long-term responders from patients who relapsed [51].

In summary, the available studies evaluating the role of cross-sectional imaging during immunotherapy in NSCLC revealed no clear advantage of [^18^F]FDG PET over CT. However, the only comparative study conducted on the role of [^18^F]FDG PET vs. CT in the neoadjuvant setting following chemo-immunotherapy, suggested that [^18^F]FDG PET predicts long-term responses better than CT. Further prospective studies are required to confirm this hypothesis.

## Discussion

In the era of precision medicine, indications to imaging tests and imaging response criteria need to be increasingly accurate and constantly scrutinized. The advent of modern anticancer therapies challenged both morphological and molecular imaging with the appearance of novel response patterns which prompted modifications of established imaging response criteria [15, 17, 52–54]. In this evolving scenario, tumor-specific response assessment frameworks should be developed, integrating information on relevant clinical questions to answer, expected treatment-induced biological effects and, in case of PET imaging, mechanisms of radiopharmaceutical cellular uptake [55].

ICIs are arguably the most successful therapies that have been introduced in the last decade and are increasingly used in several tumor types. ICIs induce self-immune responses against tumors which may have a differential impact on the readout of CT and [^18^F]FDG PET. Therefore, asking which imaging modality is best and when it should be performed during treatment with ICIs is a relevant question.

In the present paper, we showed a gap between the recommendations on the use of [^18^F]FDG PET in patients with solid tumors undergoing immunotherapy released jointly by international nuclear medicine societies [18] and the guidelines of major oncology societies [21–28], particularly those of melanoma and NSCLC. First of all, oncological societies have not developed specific recommendations for monitoring and response assessment of immunotherapy and generally consider all anticancer therapies together. As mentioned, this represents a limitation of current guidelines, as clear indications on how to use imaging diagnostic tests effectively will be increasingly required to establish imaging endpoints that correlate more closely with meaningful clinical outcomes.

While [^18^F]FDG PET is mentioned as an additional imaging modality for response assessment in melanoma, the gap becomes even more evident when considering the use of [^18^F]FDG PET for response assessment and treatment monitoring in NSCLC patients, for whom the routine use of PET is not explicitly recommended by oncological guidelines [25–27]. As illustrated in Table 2, we conducted a systematic review on the comparison between [^18^F]FDG PET and CT in the early and late response assessment to immunotherapy in patients with melanoma and NSCLC. We showed that the available clinical data derive mostly from single-institutional, small retrospective studies and do not allow to conclude on the superiority of one imaging modality over the other. Yet this would be difficult to establish, as imaging tests are only indirectly correlated with treatment outcomes. The lack of phase 3 clinical trials is often cited as an argument for questioning the adoption of PET in oncology guidelines [56]. However, the lack of well-powered randomized phase 3 studies on the role of imaging for response assessment and for the development of response-adaptive therapies is a general problem which is not limited to the context of immunotherapy and concerns both [^18^F]FDG PET and CT imaging [56, 57]. Nevertheless, CT is generally adopted as the reference imaging standard for response assessment because of historical and logistic reasons whose discussion goes beyond the scope of the present paper. It follows that, to be formally adopted in guidelines, [^18^F]FDG PET needs to demonstrate superior diagnostic accuracy than CT as well as a significant impact on clinical decision making [58]. Another critical aspect to consider when developing health interventions and clinical guidelines is cost-effectiveness, which is also very difficult to establish for diagnostic tests [59]. At present, there is no conclusive evidence of the cost-effectiveness of [^18^F]FDG PET for treatment monitoring in oncology [57] and, to our knowledge, no studies on the cost-effectiveness of [^18^F]FDG PET for monitoring immunotherapies have been published so far.

**Fig. 2:**
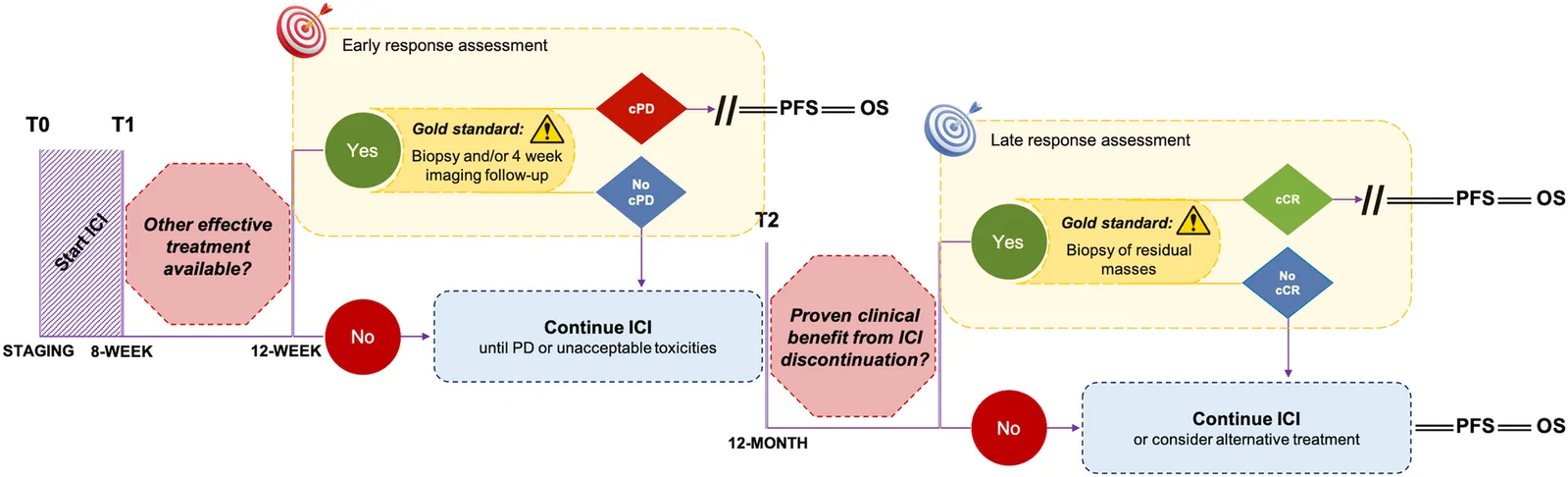
Flowchart for clinical decision making and evaluation of imaging modalities in patients undergoing immunotherapy. The figure shows how imaging may be functional to define the treatment strategy at different stages of patient management under active immunotherapy. On the left, the box represents the setting of early response assessment, between 8 and 12 weeks after the start of immunotherapy (T1). Proven evidence of the efficacy of an alternative treatment option in patients progressive under immunotherapy provides the necessary rationale for the application of imaging in this setting. If an alternative treatment option is not available, early imaging has limited clinical utility, as patients would typically continue immunotherapy until unacceptable toxicity/clinical disease progression. A theoretical clinical trial comparing two imaging modalities at this stage would need to assess which modality better identifies true progressions against the gold standard (histology or short-term imaging follow up). The box on the right represents the setting of late response assessment, e.g. one year after the start of immunotherapy (T2). At this time point, in order to safely withdraw patients from active treatment with no detrimental effects on survival outcomes, proven evidence of no additional clinical benefit of continuing immunotherapy is required, and the priority of any diagnostic test would be to identify true responders. A theoretical clinical trial comparing two imaging modalities at this stage should be designed to assess which modality better identifies true responders against the histological gold standard. A similar concept applies to the setting of treatment evaluation during or at the end of neoadjuvant treatment. The warning signs indicate a major bottleneck of trials assessing the diagnostic role of imaging, i.e. the need for validating imaging results against a histological gold standard, which is often not feasible or clinically indicated. In metastatic patients receiving adjuvant therapy, biopsy may be contraindicated because of poor clinical status or multiple sites of disease, while in late response assessment (and/or neoadjuvant settings), microscopic residual disease below imaging resolution may preclude accurate tumor sampling. Note: cCR = confirmed complete response; cPD = confirmed progressive disease; ICI = immune checkpoint inhibitors; OS = overall survival; PD = progressive disease; PFS = progression-free survival

Figure 2 illustrates a roadmap for the evaluation of the role of imaging during immunotherapy, highlighting how the identification of true progression and of true response could inform different response-adapted strategies depending on the timing of imaging during treatment.

A weakness of [^18^F]FDG PET in the context of response assessment to immunotherapy may be the potentially higher incidence of pseudoprogression compared to CT [17, 30]. Previous studies have reported the occurrence of pseudoprogression in approximately 6% across all tumor types on conventional imaging [10], whereas it may be seen in up to 17% of cases on [^18^F]FDG PET [60]. This is well explained by the fact that [^18^F]FDG is actively taken up by sites of infection/inflammation, particularly by neutrophils and monocytes/macrophages, owing to the expression of high levels of glucose transporters and to the high hexokinase activity of these cells. Given that the uptake mechanisms of [^18^F]FDG and the biology of pseudoprogression itself would lead to a high false positive rate, one may argue that the implementation of [^18^F]FDG PET in this setting probably lacks enough rationale. Furthermore, the diagnostic value of longitudinal [^18^F]FDG PET imaging has not been evaluated by previous studies, therefore there is no sufficient evidence to suggest that monitoring suspicious pseudoprogression with repeated [^18^F]FDG PET imaging would be advantageous over doing the same longitudinal monitoring with conventional CT. However, when applied later during the treatment course (i.e., after 8 weeks), in most cases [^18^F]FDG PET shows substantial agreement with CT for outcome prediction [31, 48]. Thus, the routine use of [^18^F]FDG PET earlier than 8 weeks after treatment initiation in melanoma and NSCLC cannot be indicated based on currently available evidence. At a later time-point, [^18^F]FDG PET could be considered, although it does not appear to provide additional diagnostic and prognostic information compared to CT.

On the other hand, two clinical settings have been identified in which [^18^F]FDG PET imaging, due to its high negative predictive value, proved to be prognostically superior to CT-based evaluation [32–35, 51] and pathological assessment [40], respectively, suggesting a potentially significant impact on patient care. The first scenario, which is also mentioned in the nuclear medicine recommendations [18], is represented by patients who require treatment discontinuation [32–35], where the ability to correctly identify responders could help prevent overtreatment and guide the optimal timing for immunotherapy cessation. This is a subject of ongoing debate given the lack of a clear definition of the optimal duration of ICIs treatment. In fact, while some trials mandated cessation of treatment after 2 years, others suggested continuous treatment until progression or unacceptable toxicity [61, 62].

The second setting involves patients who receive neoadjuvant immunotherapy prior to surgery, where initial evidence suggests an improved risk stratification by [^18^F]FDG PET/CT compared with CT.

Though preliminary, the available data in these two latter clinical scenarios may provide sufficient rationale for the design of randomized clinical trials investigating whether treatment discontinuation or de-escalation can be proposed to patients achieving a complete metabolic response following immunotherapy [32–35, 39, 51].

Additional elements for discussion are the interpretation criteria of [^18^F]FDG PET and its role in the detection of immune-related adverse events. As regards the first, some studies have suggested that variations of metabolic parameters relative to the whole tumor burden, such as the metabolic tumor volume and the total lesion glycolysis, may be better predictors of outcome than response criteria based on the assessment of target lesions, even applied at an earlier time point [45–50]. However, the lack of standardized methods for the extrapolation of these volumetric parameters (manual vs. semi-automatic), the variation in threshold definitions across studies, and the use of different outcomes make it challenging to compare results, and to formulate clear procedural recommendations.

Concerning immune-related adverse events, which are extensively documented in patients receiving ICI [63], it is acknowledged that [^18^F]FDG PET may allow an early detection of organ inflammation before the onset of clinical symptoms. This could help guide patient management by preventing clinical worsening and avoiding unnecessary treatment discontinuation. Emerging evidence also suggests a prognostic role for PET-detected immune organ activation [64, 65], though this finding requires prospective validation.

In summary, it appears that the recommendations for the appropriate use of [^18^F]FDG PET for monitoring immunotherapy in solid tumors [18], do not fully align with major oncological guidelines. It cannot be emphasized enough that, to fill the gap between international recommendations, efforts should be made to identify the tumor-specific settings where [^18^F]FDG PET imaging may provide additional value over conventional CT monitoring, as ICIs are becoming the standard treatment for several malignancies other than melanoma and NSCLC [66], and only an evidence-based approach will ensure the optimal allocation of resources and prevent unnecessary burden on patients and healthcare systems. In this complex scenario, the implementation and appropriate use of imaging techniques require a tailored approach aligned with the specific therapeutic strategy.

## Data Availability

No datasets were generated in this study.
